# Long noncoding RNA Braveheart promotes cardiogenic differentiation of mesenchymal stem cells in vitro

**DOI:** 10.1186/s13287-016-0454-5

**Published:** 2017-01-17

**Authors:** Jingying Hou, Huibao Long, Changqing Zhou, Shaoxin Zheng, Hao Wu, Tianzhu Guo, Quanhua Wu, Tingting Zhong, Tong Wang

**Affiliations:** 1 Guangdong Provincial Key Laboratory of Malignant Tumor Epigenetics and Gene Regulation, the Sun Yat-sen Memorial Hospital of Sun Yat-sen University, 107 Yanjiang Xi Road, Guangzhou, Guangdong 510120 China; 2 Guangdong Province Key Laboratory of Arrhythmia and Electrophysiology, 107 Yanjiang Xi Road, Guangzhou, Guangdong 510120 China; 3 Department of Emergency, the Sun Yat-sen Memorial Hospital of Sun Yat-sen University, 107 Yanjiang Xi Road, Guangzhou, Guangdong 510120 China

**Keywords:** Long noncoding RNA Braveheart, Mesenchymal stem cells, Cardiogenic differentiation, Cardiac specific transcription factors, Epithelial-mesenchymal transition, Mesoderm posterior1

## Abstract

**Background:**

Mesenchymal stem cells (MSCs) have limited potential of cardiogenic differentiation. In this study, we investigated the influence of long noncoding RNA Braveheart (lncRNA-Bvht) on cardiogenic differentiation of MSCs in vitro.

**Methods:**

MSCs were obtained from C57BL/6 mice and cultured in vitro. Cells were divided into three groups: blank control, null vector control, and lncRNA-Bvht. All three groups experienced exposure to hypoxia (1% O_2_) and serum deprivation for 24 h, and 24 h of reoxygenation (20% O_2_). Cardiogenic differentiation was induced using 5-AZA for another 24 h. Normoxia (20% O_2_) was applied as a negative control during the whole process. Cardiogenic differentiation was assessed, and expressions of cardiac-specific transcription factors and epithelial-mesenchymal transition (EMT)-associated biomarkers were detected. Anti-mesoderm posterior1 (Mesp1) siRNA was transfected in order to block its expression, and relevant downstream molecules were examined.

**Results:**

Compared with the blank control and null vector control groups, the lncRNA-Bvht group presented a higher percentage of differentiated cells of the cardiogenic phenotype in vitro both under the normal condition and after hypoxia/re-oxygenation. There was an increased level of cTnT and α-SA, and cardiac-specific transcription factors including Nkx2.5, Gata4, Gata6, and Isl-1 were significantly upregulated (*P* < 0.01). Expressions of EMT-associated genes including Snail, Twist and N-cadherin were much higher (*P* < 0.01). Mesp1 exhibited a distinct augmentation following lncRNA-Bvht transfection. Expressions of relevant cardiac-specific transcription factors and EMT-associated genes all presented a converse alteration in the condition of Mesp1 inhibition prior to lncRNA-Bvht transfection.

**Conclusion:**

lncRNA-Bvht could efficiently promote MSCs transdifferentation into cells with the cardiogenic phenotype in vitro. It might function via enhancing the expressions of cardiac-specific transcription factors and EMT-associated genes. Mesp1 could be a pivotal intermediary in the procedure.

## Background

Cardiovascular disease remains a major cause of morbidity and mortality worldwide [1]. The current treatment options for end-stage heart failure fail to regenerate myocardium that has gone through necrosis or apoptosis. Induction of cardiac regeneration to replace the lost cardiomyocytes in the injured heart represents a promising therapeutic approach in this context [2]. Stem cell therapy has emerged as a novel strategy for the treatment of ischemic heart disease during the past decade. Various stem cell types have been used for the repair of the damaged heart [2–4]. Noteworthy benefits are revealed in the regeneration of cardiomocytes following the transplantation of the precursor cells [2–4]. However, the underlying molecular mechanisms that lead to cardiomyocyte regeneration after cell therapy have not been fully elucidated.

Bone marrow-derived mesenchymal stem cells (BMMSCs) have a great potential of proliferation and differentiation, and they have been considered as a suitable source for cell therapy [5, 6]. Mesenchymal stem cells (MSCs) are capable of differentiating into cardiomyocytes under appropriate conditions both in vitro and in vivo [6]. In spite of this, the transdifferentiation efficiency of these cells is extremely low. Currently, several measures have been developed to promote the differentiation of MSCs into cardiomyocytes [7, 8]. However, most of these methods are inefficient and only a small percentage of differentiated cells can be produced. How to gain a high rate of cardiogenic differentiation from MSCs has become an issue that needs to be addressed.

Stem cell transdifferentiation into cardiomyocytes fundamentally relies on elaborate cellular and molecular mechanisms [9]. Recent discoveries demonstrate that the non-coding portion of the genome plays a crucial role in controlling cellular fate, phenotype and behavior [10]. A large number of noncoding RNAs (ncRNAs) that function as central orchestrators of cell-specific gene networks have been identified [10, 11]. An important subclass of these ncRNAs is the long noncoding RNAs (lncRNAs) that are broadly defined as regulatory noncoding transcripts more than 200 nucleotides in length. Although their biological roles and mechanisms of function remain largely elusive, accumulating evidence shows that lncRNAs participate in a wide spectrum of biological processes including cellular development, disease etiology, stem cell pluripotency and lineage specification [12]. There are already a handful reports indicating that lncRNAs can modulate cardiac differentiation during heart development [13, 14]. The long noncoding RNA Braveheart (lncRNA-Bvht) is a heart-associated lncRNA that has been identified as a pivotal regulator of cardiac lineage specification and differentiation [14]. It mediates cardiac commitment epigenetically and performs critical roles during cardiac differentiation in mouse embryonic stem cells (ESCs).

Epithelial-mesenchymal transition (EMT) is a biological process that is implicated in the developmental stage, organogenesis, tissue repair and pathological conditions [15]. Emerging evidence indicates that EMT might result in transformation of stem cell phenotypes. EMT accompanies transitions between stem-like cells and their more differentiated progeny, which perform critical functions in tissue repair and regeneration [16]. It has been revealed that EMT is involved in cardiac differentiation of ESCs and pluripotent stem cells (PSCs) [17, 18].

Mesoderm posterior 1 (Mesp1) is an essential transcription factor that marks a common multipotent cardiovascular progenitor [14]. Its expression can induce cardiovascular progenitor cells [19]. lncRNA-Bvht functions via Mesp1 to modulate the expression of cardiac transcription factors and further promote cardiogenic differentiation of ESCs [14]. Previous data show that Mesp1 is capable of initiating the EMT process by regulating EMT-associated genes [20].

In this study, lncRNA-Bvht was transfected into MSCs of C57BL/6 mice in order to investigate its implication on cardiogenic differentiation of these cells, and the underlying mechanism involved were explored in the procedure.

## Methods

### Ethics statement

Three-week-old C57BL/6 mice were obtained from the Animal Experimental Center of the Sun Yat-sen University. All animal handling and procedures were performed in accordance with protocols approved by the Animal Ethics Committee of Sun Yat-sen University (201210016).

### Isolation and culture of bone marrow-derived mesenchymal stem cells

All experiment protocols described were approved by the Institutional Animal Care & Use Committee (IACUC) at Sun Yat-sen University. Bone marrow cells were collected from 3 to 4 weeks old C57BL/6 mice by flushing femurs and tibias under aseptic conditions. Cells were cultured (37 °C, 5% CO_2_) in 25 cm^2^ culture flasks with complete culture medium supplemented with 10% fetal bovine serum, l-glutamine (4.0 mM), penicillin (100 IU/mL) and streptomycin (100 μg/mL). On the third day of culture, the medium was replaced and non-adherent cells were removed. The adherent cells were washed two times gently with phosphate-buffered saline (PBS) to reduce the degree of hematopoietic lineage cell contamination. The cells were cultured in complete culture medium and the medium was changed every 3 to 4 days for 3–4 weeks. Adherent cells gaining 90% confluence were trypsinized with 0.25% trypsin–ethylenediamine tetraacetic acid (Invitrogen) and passaged into new flasks for further expansion. Characteristics of MSCs were identified by fluorescence-activated cell sorting as previously reported [21].

### lncRNA-Bvht vector construction

The pre-lncRNA-Bvht oligonucleotides were chemically synthesized by Jinweizhi Co. Ltd. (Jiangsu, China). The primers were as follows: XhoI forward: 5′ccgCTCGAGGATCTCTGCCCCTCAGAGTCC3′, BamHI reverse: 5′cgcGGATCCAACATTTATTTTTAAAGTTTA 3′. The recovered polymerase chain reaction (PCR) products with the precursor sequence for lncRNA-Bvht were inserted into pLVX-IRES-ZsGreen1 vector. After the pre-lncRNA-Bvht viral-based vector was transformed to DH5α cells, antibiotic-resistant colonies were selected on LB-ampicillin (100 μg/mL) agar plates. The plasmid containing the target gene was verified by PCR, double digestion and DNA sequencing.

### lncRNA-Bvht transfection

The monolayer of MSCs of uniform growth attaining 90% confluence were passaged. Culture medium was removed and cells were trypsinized with 0.25% trypsin–ethylenediamine tetraacetic acid (Invitrogen). The cells were re-seeded at a density of 1 × 10^6^ cells per cell culture flask with complete medium for 24 h. Cells gaining 70–80% confluence were applied for transfection. The pLVX-IRES-ZsGreen1 vector encoding lncRNA-Bvht was transfected into MSCs with lipofectamine 2000 (Invitrogen) according to the manufacturer’s instructions. The medium was changed with fresh complete DMEM 8 h after transfection. The expression of ZsGreen was checked after 48 h of transfection.

### siRNAs experiments

MSCs were incubated at 1 × 10^6^ cells per well in six-well plates at day 0 with siRNAs against Mesp1 (Sigma) or control siRNAs (negative control, NC; Sigma). Transfection of siRNAs was performed using lipofectamine 2000 (Invitrogen) according to the manufacturer’s instructions. Mesp1 knockdown was determined by quantitative real-time PCR.

### Hypoxia/reoxygenation treatment of MSCs

MSCs in the blank control, null vector control and lncRNA-Bvht groups all experienced hypoxia/reoxygenation treatment. Cells in the different groups were incubated in serum-free media with 1% O_2_ in a Galaxy® 48 R incubator (Eppendorf/Galaxy Corporation, USA) at 37 °C for 24 h and exposed to normoxic condition (20% O_2_) for another 24 h. Normoxia was used as a negative control during the experiments for the three groups.

### Cardiogenic differentiation of MSCs

Differentiation of MSCs to cardiogenic cells was accomplished afterwards. MSCs of the three groups were seeded into six-well plates at a concentration of 1 × 10^6^ cells per well. To induce cell differentiation, the cells were incubated in a medium containing 5-AZA (10uM; Sigma–Aldrich) for 24 h at 37 °C in a humidified atmosphere with 5% CO_2_. The cells were then washed twice and the medium was replaced with normal DMEM. The medium was changed every 3 days and this procedure was terminated at 2 weeks. The morphological changes in MSCs were observed under a microscope (Olympus, CX41).

### Immunofluorescence staining

Slides with the treated cell samples taken from dishes were used directly. After drying at room temperature for a few minutes, they were permeabilized in 2% formaldehyde/PBS for 10 min. Antigen retrieval was followed by microwaving sections in sodium citrate buffer (1 M, pH 6.1). Sections were blocked with 5% bovine serum albumin (BSA) at room temperature before incubating with primary antibodies at 4 °C overnight (dilution cTnT, 1:100; α-SA, 1:100). After washing, sections were incubated with appropriate secondary antibodies and slides were counterstained with 4-6-diamidino-2-phenylindole (DAPI). Images were taken by fluorescent microscopy (Leica, Germany) with a CCD camera (Tokyo, Japan). The percentage of cTnT-positive cells was used to evaluate the efficiency of MSCs transdifferentiated into cells with the cardiogenic phenotype.

### Western blot analysis

Protein levels were measured by western blot. Cells were washed several times with PBS before collection and lysed with modified RIPA buffer. Cells were completely lysed after repeated vortexing, and supernatants were acquired though centrifugation at 14,000 × *g* for 20 min. Proteins were resolved by sodium dodecyl sulfatepolyacrylamide gel (SDS-PAGE) and transferred to a polyvinylidenedifluoride (PVDF) membrane (IPVH00010, Millpore, Boston, USA) before incubation with the primary antibodies overnight at 4 °C. The membranes were subjected to three 5-min washes with TBST and incubated with anti-IgG horseradish peroxidase–conjugated secondary antibody (Southern biotech, Birmingham, USA) for 60 min at room temperature. After extensive washing, bands were detected by enhanced chemiluminescence. The band intensities were quantified by using image software (image J 2×, version 2.1.4.7).

### Quantitative real-time PCR

Total RNA was isolated from cells using a Trizol reagent (Invitrogen) followed by digestion with RNase-free DNase (Promega). Concentration and integrity of total RNA were estimated and the real-time PCR was conducted on an ABI PRISM® 7500 Sequence Detection System using SYBR Green qPCR SuperMix (Invitrogen). The primers are described in Table 1. Specific products were amplified and detected at 95 °C for 10 min, followed by 40 cycles at 95 °C for 15 s and at 60 °C for 30 s, at which point data were acquired. The relative level of mRNA was calculated using the 2^−ΔΔCt^ method. For the assays of the molecules examined, the results were quantified as the threshold cycle of each target gene and normalized into ΔCt value. Quantifications of fold-change in gene expressions were also performed using the 2^−ΔΔCt^ method.

**Table 1:** List of primers for quantitative real-time polymerase chain reaction

Genes	Forward	Reverse
cTnT	5′- CAAAGATGCTGAAGAAGGTC-3′	5′- GATCAGAGTCTGTAGCTCATTC-3′
α-SA	5′- GATTAATGTTGCTGTTACCC-3′	5′- CCATGCTCTGTGAAATAATC-3′
Gata4	5′- CAAAGCAGCCTTGGTGACTA-3′	5′- AGAAAGTCCCAGAGCCAGGTA-3′
Gata6	5′- TTGCGGGCTCTATATGAAA-3′	5′- GCTTGTGTAGAAGGAGAAGT-3′
Nkx2.5	5′- CTTTAGGAGAAGGGCGATGA-3′	5′- GGATGGATCGGAGAAAGGT-3′
Isl-1	5′- GCTGTTTCTATATTGGTCAC-3′	5′- GCTTAAGAGACCCAGAATTT-3′
Snail	5′- TCTGCACGACCTGTGGAAAG-3′	5′- TTGGAGCGGTCAGCAAAAG-3′
Twist	5′- GAGACTCTGGAGCTGGATAACT-3′	5′- CACAAACGAGTGTTCAGACTTC-3′
N-cadherin	5′- GTGGGAATCAGACGGCTAGA-3	5′- GCTGCCCTCGTAGTCAAAGA-3′
Mesp1	5′- CAGAAACAGCATCCCAGGAA-3′	5′- TTCTAGAAGAGCCAGCATGTC-3′
β-actin	5′-AGGGAAATCGTGCGTGACAT-3′	5′-GAACCGCTCATTGCCGATAG-3′

### Statistical analysis

All quantitative data are described as mean ± SD. The significance of differences among groups was determined by the analysis of variance and Scheffe’s multiple-comparison techniques. Comparisons between time-based measurements within each group were performed with analysis of variance for repeated measurements. A *P* value <0.05 was considered to be statistically significant.

## Results

### PCR amplification and sequencing of lncRNA-Bvht

**Fig. 1:**
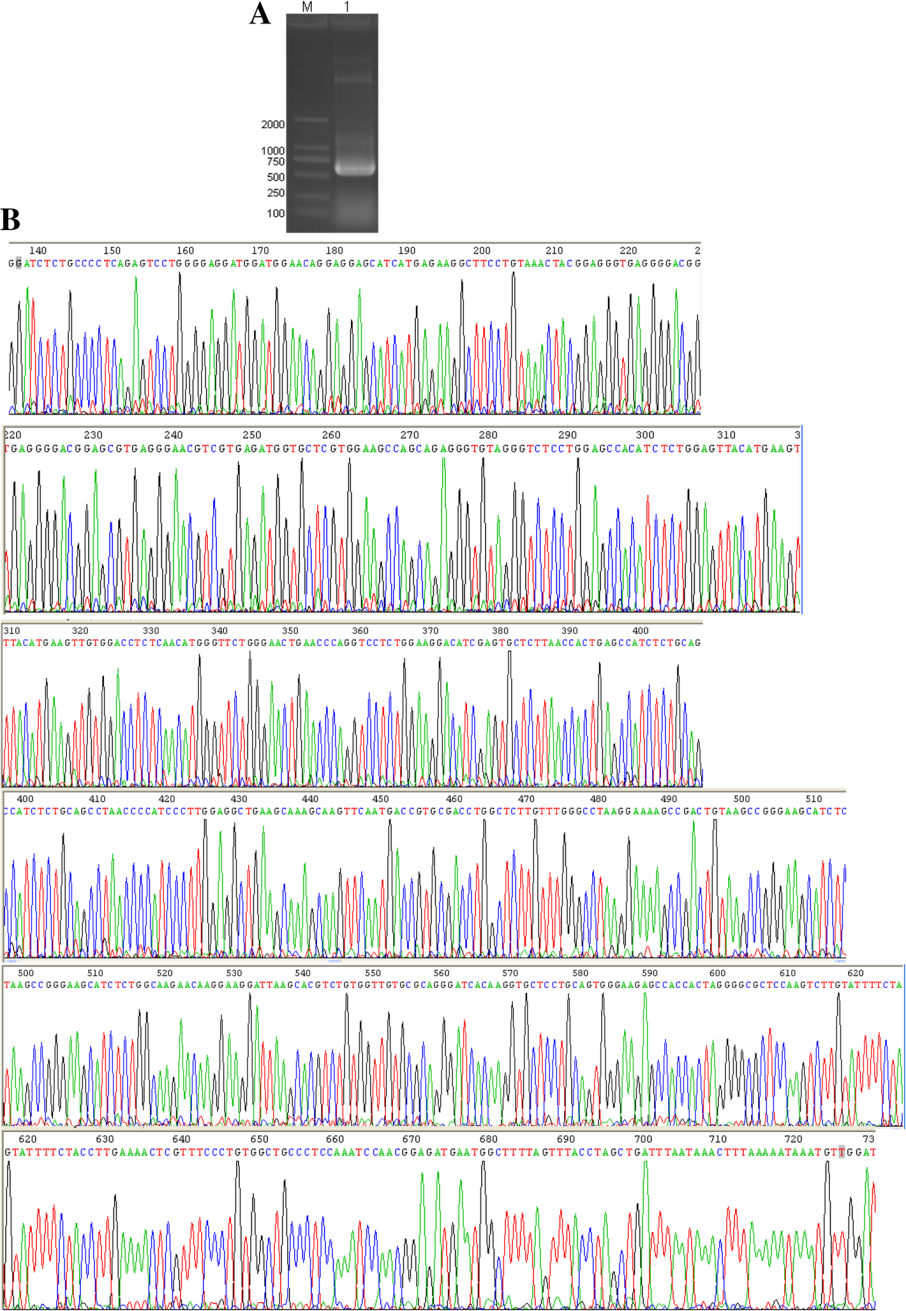
Electrophoresis of PCR products and sequence analysis of lncRNA-Bvht. **A** showed the specific band of lncRNA-Bvht at 500 bp by electrophoresis; (**B**) showed that the lncRNA-Bvht sequence was correctly constructed

DNA fragments of lncRNA-Bvht were successfully amplified by PCR. Electrophoresis revealed the specific band of lncRNA-Bvht at 500 bp (Fig. 1A). The sequence of lncRNA-Bvht was analyzed (Fig. 1B).

### lncRNA-Bvht transfection efficiency

**Fig. 2:**
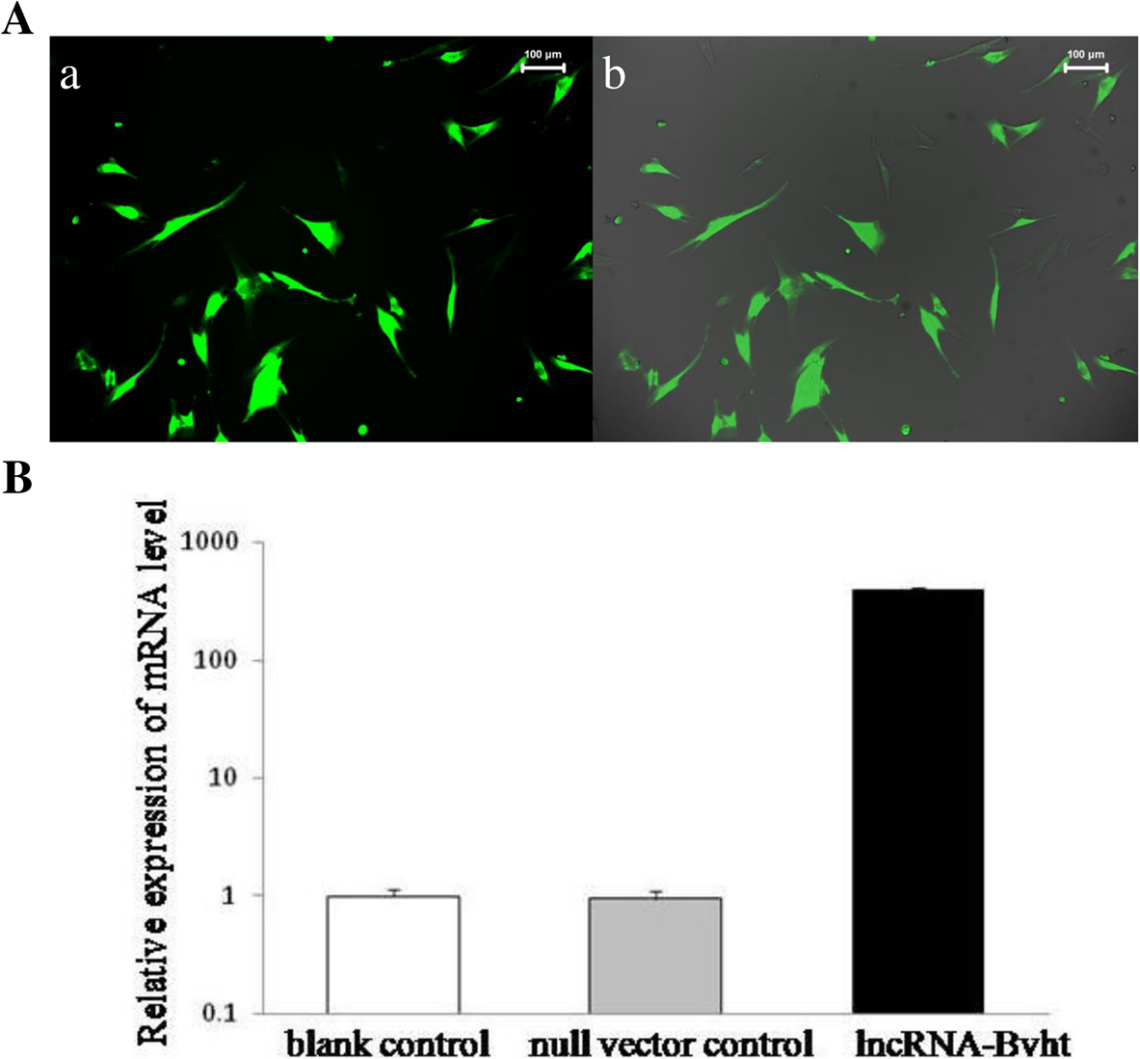
Detection of lncRNA-Bvht transfection efficiency. lncRNA-Bvht transfection efficiency was detected by the expression of ZsGreen and mRNA level of lncRNA-Bvht. **A** MSCs expressing ZsGreen after lncRNA-Bvht transfection were shown by fluorescent microscopy (×400); **a** represented MSCs transfected with lncRNA-Bvht and **b** showed that all the cells with ZsGreen expression were obtained. **B** The expression of lncRNA-Bvht in different cell groups was detected by quantitative real-time PCR

ZsGreen was expressed after MSCs were transduced with the pLVX-IRES-ZsGreen1 vector. All the MSCs with ZsGreen expression were observed under the microscope (Fig. 2A). After lncRNA-Bvht tranfection, its expression in different cell groups was detected by quantitative real-time PCR. The mRNA level was significantly higher in the lncRNA-Bvht group compared with the blank control and null vector control groups (Fig. 2B; *P* < 0.01).

### Cardiogenic differentiation in different cell groups

**Fig. 3:**
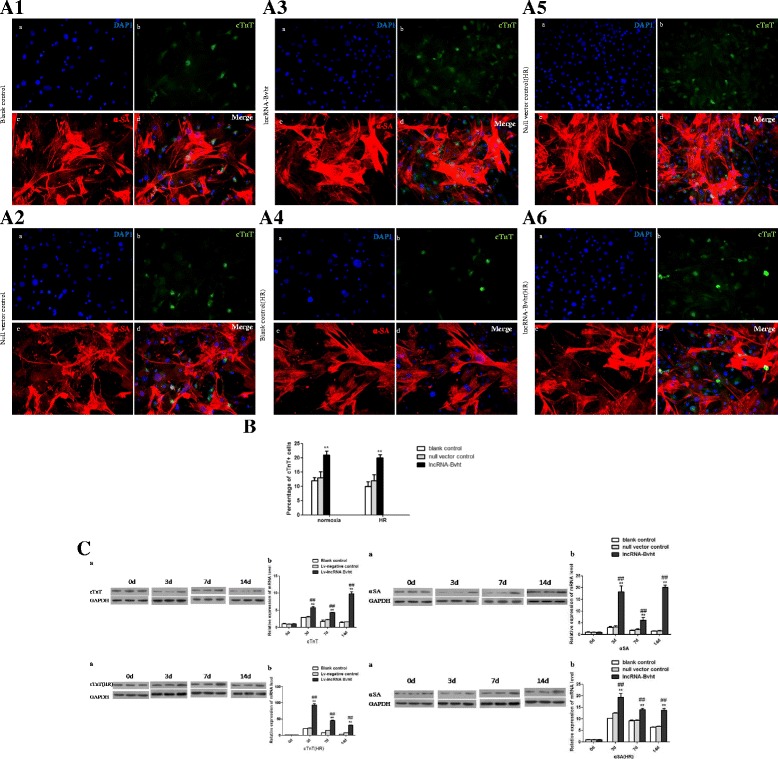
Cardiogenic differentiation in the different cell groups. Cardiogenic differentiation of MSCs in the different cell groups was evaluated by the expressions of cTnT and α-SA. **A** Confocal microscopy of immunofluorescent staining of DAPI-labeled MSCs induced by 5-AZA after 14 days (200×). Cells stained with antibody to cTnT appeared *green*, and cells stained with antibody to α-SA appeared *red*. A1–A3 represented the expressions of cTnT and α-SA under normoxic condition, and A4–A6 represented the expressions of cTnT and α-SA under the hypoxia/reoxygenation (*HR*) condition; A1 and A4, A2 and A5, and A3 and A6 represented the blank control group, null vector control group, and lncRNA-Bvht group respectively. **a** Cells derived from DAPI-labeled MSCs induced by 5-AZA displayed blue nuclei; **(b)**﻿ ﻿Cells﻿﻿ stained with antibody to cTnT appeared green in A1-A6; **(c)** Cells﻿ stained with antibody to α-SA appeared red in A1-A﻿6; **(d)** Merged image of **a**, **b**, and **c**. **B** Comparison of the percentage of cTnT-positive cells among the different groups under normoxic condition and after hypoxia/reoxygenation respectively. **C** Western blot (**a**) and quantitative real-time PCR (**b**) analysis of the expressions of cTnT and α-SA in the different cell groups. **P <0.01 , versus blank control; ^##^ *P* <0.01 , versus null vector control

Cardiogenic differentiation in different cell groups was examined by immunofluorescence staining (Fig. 3). Morphology changes could be observed in different cells groups after 14 days of induction. The differentiated MSCs expressed cardiomyocyte-specific cell markers including cTnT (green colour; Fig. 3A1–A6, images b) and α-SA (red colour; Fig. 3A1–A6, images c). The lncRNA-Bvht group (Fig. 3A3 and A6) showed an obviously higher percentage of cTnT-positive cells than the blank control (Fig. 3A1 and A4) and null vector control groups (Fig. 3A2 and A5) both under the normal condition and after the hypoxia/reoxygenation treatment (*P* < 0.01; Fig. 3A1–A6 and B); mRNA and protein levels of cTnT and α-SA were also elevated after lncRNA-Bvht transfection (Fig. 3C).

### Expressions of cardiac-specific transcription factors and EMT-associated genes in different cell groups after the induction of MSCs differentiation

**Fig. 4:**
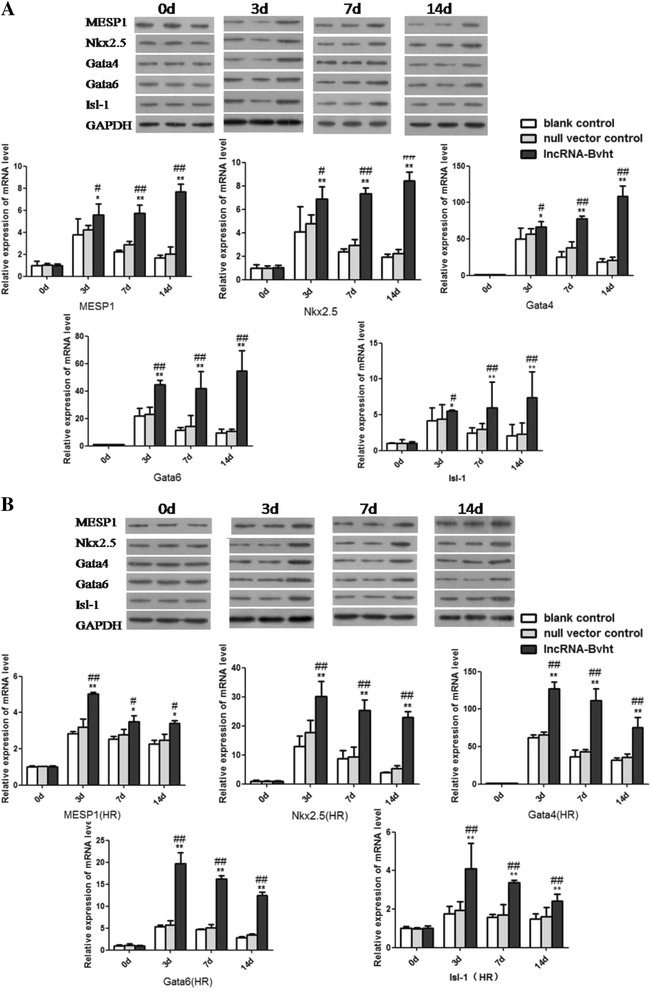
Expressions of cardiac-specific transcription factors in different cell groups after the induction of cardiogenic differentiation. **A** Expressions of cardiac-specific transcription factors in different cell groups under normoxic condition. **B** Expressions of cardiac-specific transcription factors in different cell groups after hypoxia/reoxygenation (*HR*). * *P*<0.05, ** *P*<0.01, vesu﻿s blank con﻿trol; # *P*<0.05, *#﻿# P*<0.01, vesus null vector co﻿ntrol

**Fig. 5:**
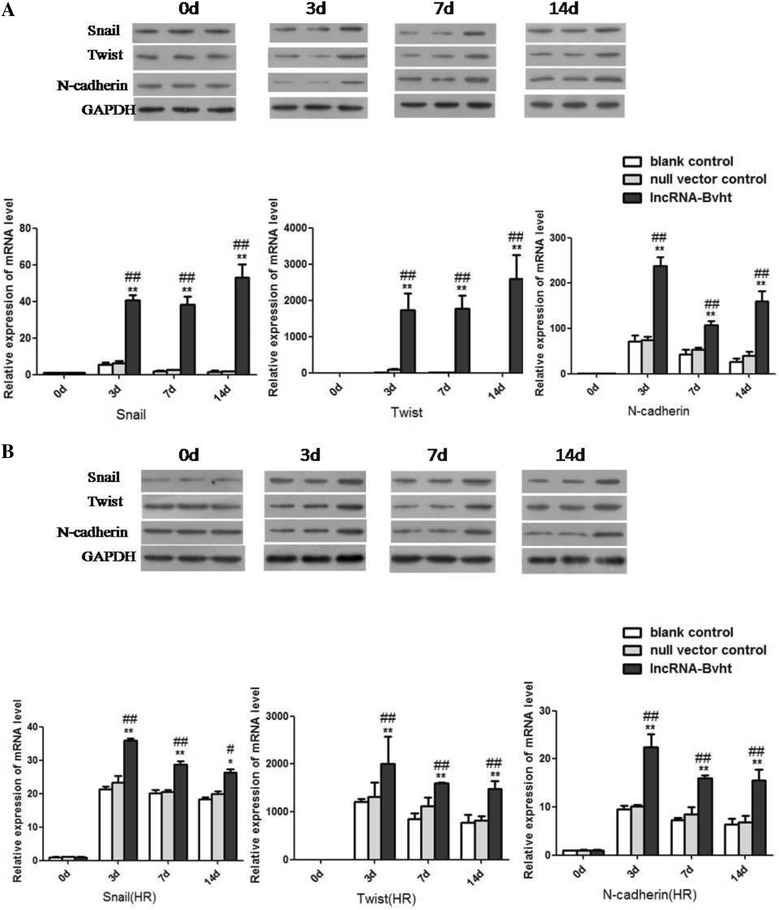
Expressions of EMT-associated genes in different cell groups after the induction of cardiogenic differentiation. **A** Expressions of EMT-associated genes in different cell groups under normoxic condition. **B** Expressions of EMT-associated genes in different cell groups after hypoxia/reoxygenation (*HR*). ﻿* *P*<0.05,** *P*<0.01, vesu﻿s blank control; # *P*<0.05,## *P*<0.01, ves﻿us null vector co﻿ntrol﻿

The expressions of cardiac-specific transcription factors including Mesp1, Nkx2.5, Gata4, Gata6, and Isl1 were examined at different time points after the induction of cardiogenic differentiation. They all showed remarkably higher expressions in the lncRNA-Bvht group than the blank control and null vector control groups both under the normal condition and after hypoxia/reoxygenation (*P* < 0.01; Fig. 4). Expression levels of EMT-associated genes including Snail, Twist and N-cadherin were also upregulated in lncRNA-Bvht group compared with the other two groups (*P* < 0.01; Fig. 5).

### Inhibition of Mesp1 interfered with MSCs transdifferentiation into cells with the cardiogenic phenotype induced by lncRNA-Bvht

**Fig. 6:**
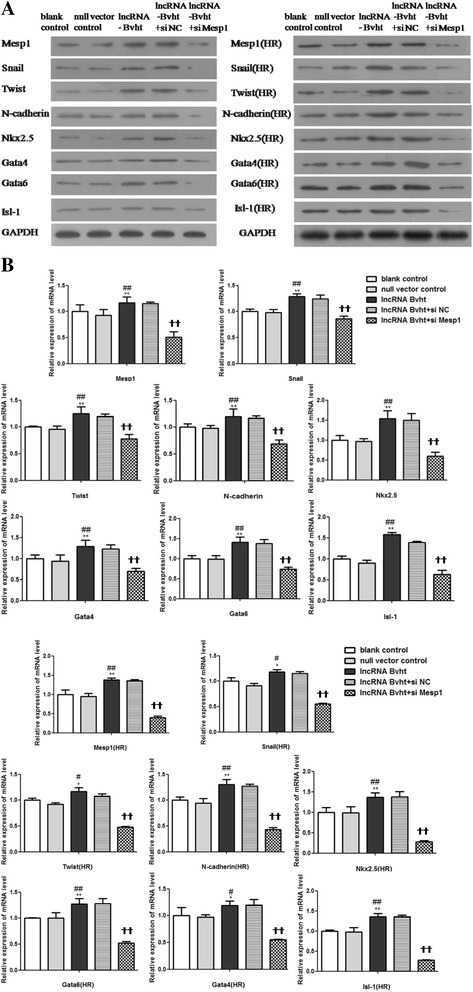
Expressions of cardiac-specific transcription factors and EMT-associated genes in different cell groups after the inhibition of Mesp1. Western blot (**A**) and quantitative real-time PCR (**B**) analysis of cardiac-specific transcription factors and EMT-associated genes in different cell groups after the inhibition of Mesp1. *HR* hypoxia/reoxygenation. *P<0.05,**﻿*P*<0.01, ve﻿su﻿s blank control; #*P*<0.05, ##*P*<0.01, ves﻿us null vector co﻿ntrol﻿; ☨*P*<0.05, ☨☨*P*<0.01, vesus lncRNA-Bvht

Anti-Mesp1 siRNAs and control siRNAs (NC) were transiently transfected into undifferentiated MSCs before lncRNA-Bvht transfection and further induction of cardiogenic differentiation. Expressions of Mesp1 and relevant downstream molecules were analyzed 72 h later. A significant reduction in Mesp1 expression was observed in the anti-Mesp1 siRNA group. Expressions of cardiac differentiation-associated genes including Nkx2.5, Gata4, Gata6, and Isl1 were all decreased, and EMT-associated genes including Snail, Twist and N-cadherin were downregulated under the condition of Mesp1 inhibition in the lncRNA-Bvht transfection group both under normoxia and after hypoxia/reoxygenation (*P* < 0.01; Fig. 6).

## Discussion

This study demonstrated that lncRNA-Bvht tranfection could efficiently promote MSCs transdifferentiation into cells with the cardiogenic phenotype in vitro. MSCs-derived cells expressed cardiac-specific markers including cTnT and α-SA. Cardiac-specific transcription factors and EMT-associated genes were upregulated following lncRNA-Bvht transfection both under normal condition and after hypoxia/reoxygenation. However, the expressions of these molecules all presented a converse alteration under the condition of Mesp1 inhibition prior to lncRNA-Bvht transfection.

To derive cardiomyocytes from stem cell precursors has been adopted as a pivotal therapeutic strategy for the repair of the injured heart. MSCs provide a valuable platform for the treatment of heart disease based on regenerative medicine [22]. Nevertheless, they show limited cardiomyogenic potential in spite of functional benefits resulting from their transplantation. Arduous efforts have been made to escalate the efficiency of cardiogenic differentiation of these cells. However, the efficacy of cellular cardiomyoplasty with MSCs remains frustrating, raising the need for alternative induction methods.

lncRNAs have been shown to be implicated in the modulation of stem cell pluripotency and cardiac differentiation [23]. It is revealed that lncRNAs are integral components of stem cell transcriptional networks [24, 25]. The knockdown or overexpression of relevant lncRNAs reciprocally influences the pluripotent transcription factors, dominating stem cell pluripotent state and lineage specificity [26–28]. Other studies have exhibited that lncRNAs regulate the cellular reprogramming process and play a pivotal role during the reprogramming of somatic cells [29, 30]. lncRNAs performing as competitive endogenous RNAs (ceRNAs) have been found to be differentially expressed in differentiating human cardiac progenitor cells (CPCs). These ceRNAs exert regulatory roles in cardiac lineage specification and differentiation [31].

Much attention has been drawn to the role of lncRNAs in heart development and cardiac differentiation [32]. Several lncRNAs have been uncovered as critical players in the development of the early cardiovascular system and cardiac differentiation [13, 14]. Some enhancer-associated lncRNAs have also been reported to take control of cardiac specification, differentiation and homeostasis [33]. lncRNA-Bvht is a newly discovered cardiac-specific lncRNA in the mouse. It promotes cardiogenic differentiation of ESCs and retains the cardiac phenotype in neonatal cardiomyocytes [14]. In this study, lncRNA-Bvht was transfected into MSCs in order to investigate its effect on cardiogenic differentiation of these cells. We discovered that a larger proportion of cells with the cardiogenic phenotype were induced after lncRNA-Bvht transfection. lncRNA-Bvht transfected MSCs displayed evenly distributed and regularly organized myofibrils after 14 days of stimuli in culture. The differentiated cells were shown to have a mature cardiogenic phenotype as evidenced by a much higher expression of cTnT. There was an enhanced level of cardiac-specific transcription factors incuding Nkx2.5, Gata4, Gata6, and Isl-1, suggesting that lncRNA-Bvht could efficiently induce the transdifferentiation of MSCs into cells with the cardiogenic phenotype in vitro by promoting the expressions of cardiac differentiation-associated genes.

Studies have demonstrated that EMT exerts a crucial role during heart development and cardiac repair. It facilitates CPCs formation during embryonic development and leads to the generation of CPCs in the adult heart [34]. EMT positively regulates cardiac differentiation of ESCs and PSCs [17, 18]. Further investigations into the EMT-based regenerative program might contribute to a more efficient strategy for cardiomyogenesis of stem cells and cardiac repair. In this study, genes implicated in EMT such as Snail and Twist were upregulated during lncRNA-Bvht induced transdifferentiation of MSCs. Twist is a crucial transcription factor that participates in the regulation of multi-progenitor states and lineage specificity of MSCs [35, 36]. It has recently been reported that Twist can modulate MSCs lineage commitment by effecting MSCs fate switching between osteogenesis and adipogenesis [36]. In this study, Twist was upregulated after lncRNA-Bvht transfaection, indicating that it might facilitate the cardiac lineage differentiation of MSCs. N-cadherin is identified as another indicator of EMT. The EMT process accompanies an elevated expression of N-cadherin. It has been revealed that N-cadherin could be a novel prospective cell surface marker of MSCs that present an apparently higher ability for cardiomyocyte differentiation [37]. N-cadherin has also been shown to be the only cell-surface gene that is highly expressed in the optimal human umbilical cord blood-derived MSCs (hUCB-MSCs). hUCB-MSCs with a notable expression of N-cadherin exhibit an enhanced cell survival rate and interact with host cardiomyocytes more effectively [38]. In this study, we also found that N-cadherin expression level was significantly enhanced after lncRNA-Bvht transfection. The aforementioned data suggest that EMT-associated genes were involved in lncRNA-Bvht mediated cardiogenic differentiation of MSCs.

Mesp1 is a basic helix-loop-helix transcription factor that symbolizes the earliest progenitors of the cardiovascular system [19]. Mesp1 positive cells have been reported to show an increased expression of mesodermal and EMT markers and preferential cardiac differentiation, implying a critical role of Mesp1 in promoting EMT and cardiac differentiation [20]. Evidence shows that Mesp1 regulates EMT and cardiovascular differentiation in ESCs and CPCs [20, 39]. Several pharmacological agents have been introduced to enhance the cardiomyogenic ability of MSCs for cardiac repair [40]. MSCs treated with these agents show an appreciably elevated expression of Mesp1, with a later widespread upregulation of the structural genes of cardiomyocytes. Other data display that a recombinant method using various growth factors and cell cytokines guides MSCs into a cardiac progenitor phenotype with a significantly increased expression of Mesp1, conferring cardiac competency on these cells to regenerate new cardiomyocytes [41]. In this study, Mesp1 was remarkably increased at different time points after the induction of cardiogenic differentiation both under the normal condition and after hypoxia/reoxygenation following the lncRNA-Bvht transfection. There was a distinct upregualtion of cardiac differentiation-associated genes and biomarkers of the EMT process. Mesp1 repression before lncRNA-Bvht transfection significantly downregulated the expressions of cardiac differentiation-associated genes and biomarkers of EMT, indicating that lncRNA-Bvht mediated upregulation of Mesp1 might contribute to the expression of cardiac differentiation-associated genes and biomarkers of the EMT process, which subsequently leads to the transdifferentiation of MSCs into cells with the cardiogenic phenotype.

### Limitations of the study

This study was performed in vitro, in vivo studies will be conducted in the future to investigate the effects of lncRNA-Bvht on cardiomyocyte differentiation of MSCs in vivo. In this work, we mainly demonstrated that lncRNA-Bvht could induce the transdifferentiation of MSCs into cells with cardiogenenic phenotypes. Further studies will be focused on functional properties of these differentiated cells, including their contraction and electrophysiological characteristics and whether they possess the ability to form functional syncytium with host cardiomyocytes. Specific signaling pathways involved in the functional mechanisms of lncRNA-Bvht will also be explored later on.

## Conclusions

lncRNA-Bvht could efficiently promote MSCs transdifferentiation into cells with the cardiogenic phenotype in vitro. It might function via enhancing the expressions of cardiac-specific transcription factors and EMT-associated genes. Mesp1 could be a crucial intermediary in the procedure. This study provides new insight for the application of lncRNAs in stem cell-mediated cardiomyocyte regeneration. Further work on the functional mechanisms of these novel molecules might be conducive for improving the therapeutic efficiency of MSCs transplantation.

## Abbreviations

ceRNA: Competitive endogenous RNA
CPC: Cardiac progenitor cell
EMT: Epithelial-mesenchymal transition
ESC: Embryonic stem cell
hUCB-MSC: Human umbilical cord blood-derived mesenchymal stem cell
lncRNA-Bvht: long noncoding RNA Braveheart
lncRNA: long noncoding RNA
Mesp1: Mesoderm posterior1
MSC: Mesenchymal stem cell
ncRNA: noncoding RNA
PBS: Phosphate-buffered saline
PSC: Pluripotent stem cell
